# The challenges and rewards of engaging a skeptical public

**DOI:** 10.1186/2045-4015-2-12

**Published:** 2013-03-27

**Authors:** Lori Uscher-Pines, Arthur L Kellermann

**Affiliations:** 1 RAND Corporation, 1200 S Hayes St, Arlington, VA, 22202, USA

## Abstract

Findings published in this issue suggest that a substantial subset of the Israeli public generally trusts government, yet is determined to make their own judgments about the need for precautionary action in certain types of public health emergencies. This reflective approach, which may be common in other countries as well, poses a substantial challenge to achieving desired levels of compliance, particularly when the threat requires swift and concerted action. The aim of this commentary is to discuss both the challenges and the rewards of engaging a public that wants to weigh evidence prior to taking action in an emergency, rather than defer to expert judgment. While engaging a skeptical public can be difficult, a reflective public acknowledges that preparedness is a shared responsibility of government and individuals and may be receptive to messages about the need for household and community self-sufficiency in a disaster. This is a commentary on the article “Analysis of Public Responses to Preparedness Policies” by Velan and colleagues.

## 

The goal of emergency risk communications is to convince the public to take certain actions to prevent or mitigate the consequences of a large scale threat to public health. Examples include emergency evacuation orders, calls to stockpile food, water, and supplies in advance of a storm, and urging members of the public to engage in social distancing to reduce the transmission of an infectious disease. The effectiveness of such messages depends in part on how well they are crafted and disseminated and in part on individuals’ willingness to adopt the desired behavior. We understand a great deal about the former, but significantly less about the latter. That is what makes the article by Velan and colleagues in this issue of the Journal both interesting and important [1].

Their paper presents the results of a telephone survey of the public’s willingness to comply with government recommendations to mitigate the risks posed by two recent threats to the health of Israel’s population: 1) acquisition (at no cost to the individual) of gas masks in 2010 to prepare for the potential for chemical warfare against civilians and 2) acceptance of influenza vaccine during the 2009 H1N1 influenza pandemic. Rather than simply document self-reported compliance rates among different subgroups of Israel’s demographically diverse population, the survey allowed the authors to probe such important issues as trust, knowledge, and varying beliefs about the benefits and potential risks of complying with government recommendations. The two scenarios – one chemical and one biological; one countermeasure promoted by the Homeland Command and the other by the Israeli Ministry of Health – also provided opportunities to examine potential differences in the public’s perceptions of trust and the importance of compliance in response to qualitatively different threats.

The value of this type of research cannot be over-stated. The public’s willingness to accept and comply with governmental recommendations in potential or actual public health emergencies is vital to ensure an effective response. In fact, it can have a major impact on morbidity and mortality [2,3]. Velan and colleagues determined that a substantial subset of the Israeli public generally trusts government, yet is determined to make their own judgments about the need for precautionary action in certain situations. Rather than automatically deferring to government authority or expert judgment, reflective individuals want to weigh the evidence for themselves prior to taking action. The challenge is that when the threat requires swift and concerted action, reflection can complicate efforts to achieve high levels of compliance.

Clearly, more must be learned about what motivates individuals to accept or reject official guidance to receive a recommended vaccine, stay home from work, or evacuate a low-lying coastal community in advance of a hurricane. Until then, policy makers and emergency response officials will struggle to identify the most effective communication strategies to reach these “trusting but reflective” individuals. Although many emergency response officials probably wish these people would simply fall into line, the fact that many citizens think through their options before taking action is not necessarily a bad thing.

## The benefits of engaging a reflective public

Conventional wisdom in emergency preparedness suggests that during a national emergency, the public should trust official government spokespersons, ignore conflicting advice from other sources, and swiftly comply with recommended actions. In certain situations, such immediate evacuation of an area threated by an impending tsunami, this makes sense. Velan and colleagues’ findings suggest, however, that in other situations (such as when the threat is less imminent or more uncertain), unity of response is unlikely. This may be particularly true now that many individuals can independently access information about a potential health threats from an array of unofficial sources, and use social media, such as Face book or Twitter, to share their opinions with others. The potential for informal information sources to undermine or even contradict official guidance is particularly great when the magnitude of the threat is uncertain and the recommended action (such as accepting a novel vaccine) involves even the slightest degree of risk [4].

As Velan and colleagues note, an added challenge of dealing with a public inclined to make reflective assessments is that their decisions may be influenced, to a large degree, by self-interest rather than broader public health benefits, such as herd immunity and reduced absenteeism. Economic considerations can trump the health risks of noncompliance. For example, in a pandemic, the American public tends to favor simplistic but ineffective actions such as closing borders, over more effective actions, such as social distancing, that require individual initiative and may impose economic hardships [3]. For public health authorities, the problems of individual-level decision biases and competing values complicate their pursuit of broad public health goals.

Although the challenges of reaching a reflective public are real, they also present potential opportunities and benefits for preparedness officials. A reflective public that actively considers evidence is one that implicitly understands that emergency preparedness and response is a shared responsibility between the government and individual citizens. The community resilience literature discusses the fundamental role that engaged citizens play in helping a population swiftly recover from a disaster [5,6]. Israel’s experience with gas mask distribution shows that unconditional acceptance of government recommendations does not ensure high levels of public compliance. Other factors, such as apathy or cost, may intervene. In fact, passivity may be more dangerous than skepticism. Apathetic individuals ignore local authorities, fail to weigh the pros and cons of recommended action, and go about their lives as if nothing will happen. Resilient communities may question or even challenge public information, but their engagement provides opportunities to identify and swiftly counter misinformation and false rumors. Apathy is harder to overcome.

Officials responsible for engaging the public in public health emergencies must learn to listen to the public and adapt their messaging to address the public’s concerns. In the past, officials considered public skepticism a nuisance, or worse, an active impediment to effective response. However, much as the myth of the panicked public that exhibits anti-social behavior in disasters has been debunked [7], the notion that a reflective public is an obstacle to preparedness may be replaced by the understanding of the value of engagement and dialog.

## Harnessing trust and reflectivity to promote self-sufficiency

The implications of this study go beyond enhancing public compliance with specific messages to broader preparedness goals, such as promoting self-sufficiency. Here, we define self-sufficiency as broadly accepting personal (or household or even community) responsibility for preparedness. It involves taking steps to ensure that you can draw on your own resources or that of your extended support network. In Israel, and perhaps in certain other countries as well, there is an understanding that citizen preparedness is a civic duty, and public messaging and engagement presupposes that ordinary citizens will assume the role of first responders. Education about self-sufficiency begins early in Israel, as many educational materials are aimed at young children [8]. Although the U.S. has not been as successful in fostering a national culture of citizen preparedness [8], its Federal Emergency Management Agency (FEMA) emphasizes the importance of personal preparedness. Because it takes time to mobilize national assets, FEMA has long warned individuals that in the event of a major disaster, they should not expect outside assistance to arrive for up to 72 hours [7,9]. Since fostering self-sufficiency is important, it is critical to learn how to engage the public to promote it.

At first glance, it would appear that the message “trust us” is undermined by the message “prepare yourself, because for the first 3 days, you’ll be on your own.” However, the relationship between trust and self-sufficiency is complex [10]. These concepts do not have to be at odds; in fact, trust and reflectivity can be harnessed to promote self-sufficiency. Velan and colleagues describe trust in terms of the authorities’ motivation to serve the public interest. Yet there are many other dimensions of trust that were not explored, further adding to the complexity of this construct. For example, beliefs that the government is competent to handle an emergency, that it will approach communication with honesty and transparency, and that the response will be fair and equitable, all capture different components of trust [11-13].

Individuals who are inclined to trust their government are more likely to follow public health directives [2]. They are also more likely to follow government recommendations to develop a household preparedness plan and stockpile an adequate cache of supplies [10]. The reflective individual acknowledges his or her role in preparedness and for this reason, he or she is more likely to understand the importance of self-reliance. As challenging as the job of risk communication in Israel appears to be, it may be even tougher in the United States. Not only is America much larger than Israel; it has a cultural tradition of distrusting government authority [14,15]. As our recent experience with the Deepwater Horizon Oil Spill showed, in a federal system such as the U.S., the large number of government agencies, at the federal, state and municipal level issuing “official guidance” almost guarantees that official messaging will be inconsistent or even contradictory [16]. In such circumstances, the public may turn to more trusted information sources such as their personal healthcare provider, a community pharmacist, or a neighbor for advice [17]. This is another reason why engaging local community members is so important. Although Velan’s team measured trust at only one point in time, other research suggests that trust fluctuates over the course of an event [2]. In light of this fact, Government authorities should track shifts in public opinion and confidence so they can modify messaging when needed, and leverage periods of high public trust to promote desired actions.

Studies of how the public thinks about emergency preparedness, response, and public information matter. They are important to help emergency response officials effectively communicate with the communities they serve. More work of this sort is needed to explore the associations between different dimensions of trust and the division of responsibilities for disaster planning and response. We commend Velan and colleagues for focusing on this topic.

Ultimately, our goal as advocates for public health and emergency preparedness should not only be to boost citizen compliance through effective guidance; we must also earn the public’s confidence through effective listening. That may be the best way to assure that Israel, the United States, and other nations are properly prepared for future disasters and acts of terrorism.

## Competing interests

The authors declare that they have no competing interests.

## Author’s information

Lori Uscher-Pines, PhD, MSc is an Associate Policy Researcher at the RAND Corporation who specializes in emergency preparedness, vaccination, and emergency care utilization and delivery.

Art Kellermann, MD, MPH, holds the Paul O’Neill-Alcoa Chair at the RAND Corporation. An emergency physician and health services researcher, he has written for years on topics ranging from violence and injury prevention to public health preparedness.

## Commentary on

Analysis of Public Responses to Preparedness Policies: The Cases of H1N1 Influenza Vaccination and Gas Masks Distribution. By Baruch Velan, Valentina Boyko, Gilead Shenhar, Liat Lerner-Geva, Giora Kaplan.
